# Upfront Surgery for c-Stage IIIB Non-Small Cell Lung Cancer with an Abnormally High Preoperative Serum Carcinoembryonic Antigen Level: A Case Report

**DOI:** 10.70352/scrj.cr.25-0707

**Published:** 2026-07-04

**Authors:** Ryuki Tsunemi, Ryusuke Sumiya, Shinsuke Uchida, Mariko Fukui, Takeshi Matsunaga, Aritoshi Hattori, Takuo Hayashi, Kazuya Takamochi, Kenji Suzuki

**Affiliations:** 1 Department of General Thoracic Surgery, Juntendo University School of Medicine, Tokyo, Japan; 2 Department of Human Pathology, Juntendo University School of Medicine, Tokyo, Japan

**Keywords:** upfront surgery, carcinoembryonic antigen, non-small cell lung cancer

## Abstract

**INTRODUCTION:**

Carcinoembryonic antigen (CEA) is one of the most widely used tumor markers in clinical practice. In non-small cell lung cancer, elevated preoperative serum CEA levels have been reported to be associated with a poor prognosis.

**CASE PRESENTATION:**

A 43-year-old man with primary lung cancer of the right upper lobe was referred to our hospital. Initial blood tests revealed a markedly elevated serum CEA level of 1160.0 ng/mL. Chest CT identified a 55-mm tumor in the right hilar region, with enlargement of the right hilar and prevascular (#3a) lymph nodes (c-T3N2M0, Stage IIIB according to the Union for International Cancer Control tumor–node–metastasis classification, 8th edition). The patient underwent a type A extended sleeve lobectomy and pulmonary vein transposition. However, due to suspected pulmonary venous return failure, a right completion pneumonectomy was performed on POD 7. The patient’s serum CEA levels returned to the normal range within 2 months postoperatively. He has remained recurrence-free for more than 4 years since the surgery.

**CONCLUSIONS:**

This case highlights that upfront surgery may be a valid treatment option in selected patients with c-Stage IIIA or IIIB disease, even in the presence of an abnormally high preoperative serum CEA level.

## Abbreviations

CEA
: carcinoembryonic antigen
NSCLC
: non-small cell lung cancer
TNM
: tumor–node–metastasis

## INTRODUCTION

CEA is one of the most widely used tumor markers in clinical practice. In NSCLC, elevated preoperative serum CEA levels have been reported to be associated with a poor prognosis.^1,2)^ The TNM classification is also an important prognostic indicator and serves as a key criterion for determining treatment strategies. Currently, the indications for surgical resection in c-Stage III NSCLC are carefully evaluated.^3,4)^

Herein, we present a case of 4-year disease-free survival following a right pneumonectomy for p-T3N2M0 Stage IIIB lung adenocarcinoma, according to the 8th edition of the Union for International Cancer Control TNM classification, with an abnormally high preoperative serum CEA level.

## CASE PRESENTATION

**Fig. 1:**
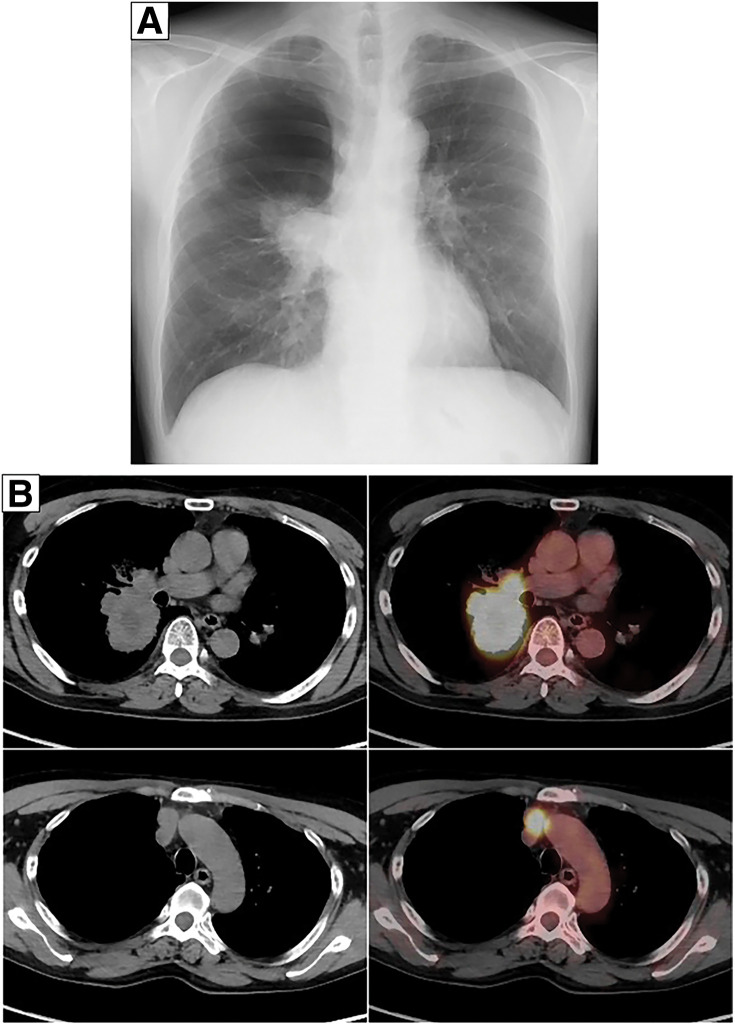
Representative radiological images. (**A**) Chest radiograph showing a mass shadow in the right hilar region. (**B**) Chest CT and fluorine-18-fluorodeoxyglucose PET showing a 55 × 42-mm mass extending from segment 2 to segment 6, with a maximum standardized uptake value of 17.20 in the upper panels. The lower panels show enlargement of the prevascular (#3a) lymph node with maximum standardized uptake values of 14.55.

**Fig. 2:**
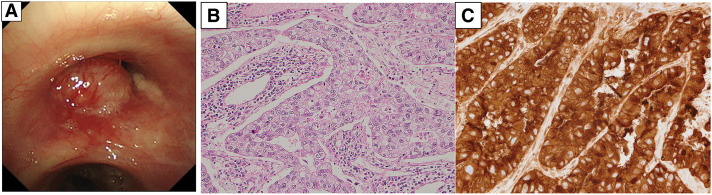
Representative images of preoperative bronchoscopy and pathological findings. (**A**) A tumor with oozing at the ostium of the right upper bronchus. (**B**) Hematoxylin and eosin staining revealed atypical cells with mildly clear cytoplasm and enlarged nuclei, proliferating in nests, tubular, and cribriform patterns (×100). (**C**) CEA immunohistochemical staining demonstrated strong positivity in the tumor cells (×200). CEA, carcinoembryonic antigen

A 43-year-old man with a history of chronic obstructive pulmonary disease and a 20-pack-year smoking history was diagnosed with primary lung cancer in the right upper lobe, with a single lymph node metastasis at station 3a. Although concurrent chemoradiotherapy was recommended, the patient was referred to our hospital due to the presence of blood-stained sputum and a preference for surgical intervention. Initial blood tests revealed a markedly elevated serum CEA level of 1160.0 ng/mL. Pulmonary function test revealed a vital capacity of 3070 mL (68.3% predicted). The forced expiratory volume in 1 s was 2180 mL, and the ratio of forced expiratory volume in 1 s to forced vital capacity was 70.1%. Chest radiography revealed a mass shadow in the right hilar region (**Fig. 1A**). Chest CT and fluorine-18-fluorodeoxyglucose PET revealed a 55 × 42-mm mass extending from segment 2 to segment 6, with a maximum standardized uptake value of 17.20 (**Fig. 1B**). These imaging modalities also revealed enlargement of the right hilar and prevascular (#3a) lymph nodes, with maximum standardized uptake values of 10.12 and 14.55, respectively (**Fig. 1B**). Contrast-enhanced brain MRI showed no evidence of brain metastasis. Bronchoscopy revealed a tumor with oozing at the ostium of the right upper bronchus (**Fig. 2A**), and biopsy confirmed poorly differentiated NSCLC (c-T3N2M0, Stage IIIB). Due to a high risk of hemoptysis, urgent surgical resection was planned.

During a multidisciplinary conference involving thoracic surgeons, medical oncologists, and radiation oncologists, we decided that the upfront surgery would be the best option because of the presumed risk of hemoptysis, single-station N2, and the feasibility of preserving the right basal segments through an extended sleeve lobectomy. The patient underwent a right upper and middle lobectomy with segmentectomy of segment 6 (a type A extended sleeve lobectomy) and mediastinal lymph node dissection. As the station 3a lymph node was suspected to invade the superior vena cava, partial resection and plication were performed. To reduce the risk of venous return obstruction due to tension on the basal segment of the pulmonary vein, pulmonary vein transposition was performed by anastomosing it to the superior pulmonary vein. Histopathological examination revealed atypical cells with mildly clear cytoplasm and enlarged nuclei, proliferating in nests, tubular, and cribriform patterns (**Fig. 2B**). Spread through air spaces was also observed. Immunohistochemical staining showed positivity for thyroid transcription factor-1 and focal positivity for p40, while staining for Napsin A and cytokeratin 5/6 was negative, findings consistent with adenocarcinoma. CEA immunohistochemical staining demonstrated strong positivity in the tumor cells (**Fig. 2C**). Although metastatic involvement was observed only in the station 3a lymph node with extranodal extension into the surrounding soft tissue, there was no pathological evidence of direct invasion of the superior vena cava. The tumor was finally diagnosed as invasive adenocarcinoma, p-T3N2 (3a) M0, Stage IIIB.

Postoperatively, chest radiographs showed decreased lucency in the residual lung, with a gradual increase in inflammatory markers. Due to suspected pulmonary venous return failure, a completion right pneumonectomy with pericardial fat flap coverage of the bronchial stump was performed on POD 7. The patient’s overall condition gradually improved following the second operation, and he was discharged 7 days later.

**Fig. 3:**
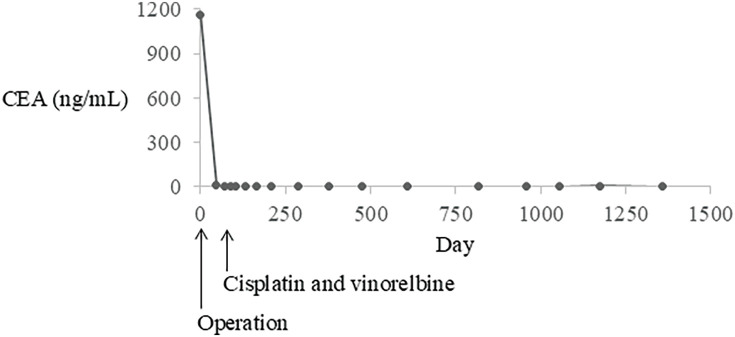
A flowchart of the clinical course. The vertical axis shows CEA levels, and the horizontal axis shows POD. Arrows denote the operation and the start date of chemotherapy. CEA, carcinoembryonic antigen

At 1 month postoperatively, the patient’s CEA level had decreased markedly to 10.5 ng/mL and normalized within 2 months. He subsequently received adjuvant chemotherapy with cisplatin and vinorelbine. His serum CEA levels remained within the normal range, and follow-up chest CT performed 4 years postoperatively showed no evidence of tumor recurrence (**Fig. 3**).

## DISCUSSION

CEA was originally reported by Gold and Freedman as a cancer antigen specific to the human digestive system.^5)^ It is one of the most widely used tumor markers in clinical practice, not only for gastrointestinal cancers but also for adenocarcinomas, including those of the lung and breast. Recently, its potential as a prognostic factor in NSCLC has been increasingly recognized.^1,2,6–10)^

Several studies have evaluated the association between preoperative serum CEA levels and survival in patients with NSCLC. Wang et al. performed a meta-analysis of 16 studies (n = 4296 patients) and concluded that preoperative serum CEA overexpression indicated a poor prognosis.^1)^ Similarly, Li et al. conducted a retrospective cohort study involving 1130 patients with NSCLC and integrated their findings with previously published studies using a cumulative meta-analysis, indicating that the pretreatment serum CEA level is an independent prognostic factor for overall and disease-free survival.^2)^ However, our patient has remained recurrence-free for more than 4 years after surgery despite an abnormally high preoperative serum CEA level of more than 1000 ng/mL. Several studies have also indicated that postoperative serum CEA levels are an important predictor of subsequent survival.^6–10)^ In our case, the CEA level rapidly decreased to 10.5 ng/mL at 1 month postoperatively and normalized within 2 months. It has remained within normal limits to date, with no evidence of recurrence on imaging. This rapid decline and subsequent normalization of postoperative serum CEA levels may reflect complete resection of the tumor tissue. This case suggests that curative surgical treatment should not be dismissed solely on the basis of high preoperative serum CEA levels.

Another notable aspect of this case is the success of surgical intervention despite a clinical diagnosis of c-T3N2M0, Stage IIIB. (This corresponds to c-T3N2aM0, Stage IIIA according to the 9th edition of the Union for International Cancer Control TNM classification.) As RCTs evaluating the role of surgery in N2 NSCLC cases have not demonstrated an overall survival benefit of surgical intervention,^11–13)^ the indications for surgical resection should be carefully evaluated. In this case, the tumor was initially considered unresectable, and concurrent chemoradiotherapy was recommended at the previous hospital. However, since the patient complained of blood-stained sputum and bronchoscopy revealed a tumor exposed in the bronchus, he was considered to be at high risk of hemoptysis if treated with the recommended therapy. Therefore, we opted for upfront surgical resection. In recent years, neoadjuvant chemotherapy combined with immune checkpoint inhibitors has become an important treatment option for locally advanced NSCLC. However, hemoptysis is an oncologic emergency and should be considered separately from long-term oncologic outcomes. Multidisciplinary conferences should be considered for patients with a high risk of oncology emergencies, such as hemoptysis.

In addition to having chronic obstructive pulmonary disease, the patient had a tumor extending from segment 2 to segment 6 in the right hilar region. Pneumonectomy is generally associated with significant morbidity and mortality, particularly after right-sided procedures.^14)^ N2 disease has also been reported to be an adverse prognostic factor after pneumonectomy for NSCLC.^15)^ Therefore, the indication for pneumonectomy should be determined cautiously through careful patient selection, and lung-sparing procedures are preferred when anatomically feasible and oncologically appropriate. Extended sleeve lobectomy is an effective procedure to avoid pneumonectomy in cases where tumors or metastatic lymph nodes extend into the hilar region and require resection of more than 1 lobe.^16,17)^ Compared to pneumonectomy, extended sleeve lobectomy has been associated with better long-term survival outcomes.^18)^ In our case, resection of the right upper lobe, middle lobe, and segment 6 was necessary. Therefore, a type A extended sleeve lobectomy and pulmonary vein transposition were performed. Although a completion right pneumonectomy was ultimately required due to postoperative failure of pulmonary venous return, the patient has made a good recovery and currently maintains an Eastern Cooperative Oncology Group performance status of 0.

Careful evaluation is essential when determining whether a case of N2 disease is resectable. While there are no absolute criteria, single-station N2 disease is sometimes considered resectable. According to a report on upfront surgery for clinical single-station N2 NSCLC, the 5-year overall survival rate for pathological single-station N2 was significantly higher than that for pathological multiple-station N2.^19)^ In our patient, metastasis to the N2 station was observed only in the prevascular lymph nodes (#3a). Although it invaded the superior vena cava, it was completely removed via partial resection and plication of the superior vena cava.

## CONCLUSIONS

Herein, we report a case of long-term survival after right pneumonectomy for lung adenocarcinoma with an abnormally high preoperative serum CEA level. This case suggests that upfront surgery may be a valid treatment option in selected patients with c-Stage IIIA or IIIB disease.

## DECLARATIONS

### Availability of data and materials

The datasets supporting the conclusions of this article are included within the article.
